# The complete mitochondrial genome of *Archamia macropterus* (Perciformes; Apogonidae) and phylogenetic studies of Perciformes

**DOI:** 10.1080/23802359.2019.1681311

**Published:** 2019-10-24

**Authors:** Yun Pan, Jian Chen, Chan Liu, Lin Zeng, Xiangjiong Zhao

**Affiliations:** a National Engineering Research Center of Marine Facilities Aquaculture, Zhejiang Ocean University, Zhoushan, People’s Republic of China;; b Bureau of Agriculture and Rural Affairs of the People’s Republic of Zhoushan, Zhoushan, Zhejiang province, People’s Republic of China

**Keywords:** *Archamia macropterus*, mitogenome, phylogenetic tree

## Abstract

The complete mitochondrial genome sequence of *Archamia macropterus* was determined. The complete mitochondrial genome was 16,513 bp in length and contained 13 protein-coding genes, 22 transfer RNA genes, 2 ribosomal RNA genes, and 2 non-coding region (the control region and the origin of light strand replication). The overall base composition was A 26.37%, T 25.61%, C 30.80%, and G 17.22%. All protein-coding genes started with an ATG initiation codon, except COI used GTG. With the exception of ND6, all other genes were encoded on the heavy strand, the NJ tree demonstrated that A. *macropterus* has a closest relationship with *Cheilodipterus quinquelineatus* and *Apogon semilineatus*.

*Archamia macropterus* (Perciformes; Apogonidae) is widely distributed in the tropical Indian and Pacific oceans, Indonesia, Philippines, Australia, Japan, China, and so on. Limited information is available on the genetic resources for this species (Phung 1998). In the present study, we determined the complete mitochondrial genome (mitogenome) of *A. macropterus* (GenBank accession number: MN066612) and constructed the phylogenetic relationship within Perciformes, which contributes to future researches on population genetic structure and taxonomic resolution.

Specimens of *A. macropterus* was obtained from the South China Sea (18°36′39″N; 113°17′43″E) and stored in the laboratory of Zhejiang Ocean University with accession number 20180921JDS26. Total genomic DNA was extracted from muscle tissue by the phenol-chloroform method according to Barnett and Larson (2012) and then used for PCR amplification and sequencing. The phylogenetic tree was conducted based on the neighbor-joining (NJ) method using MEGA5 (Tamura et al. 2011).

Similar to the typical mitogenome of vertebrates (Thacker and Roje 2009; Zeng et al. 2016), the complete mitochondrial genome of *A. macropterus* was 16,513 bp in length, consisting of 13 protein-coding genes, 22 transfer RNA genes (tRNA), 2 ribosomal RNA genes (12SrRNA and 16SrRNA), a putative control region, and one replication origin. The overall base composition is A 26.37%, T 25.61%, C 30.80%, and G 17.22%. A + T content (51.98%) was slightly higher than the G + C content. Twelve protein-coding genes (ND1, ND2, COXI, COXII, ATP8, ATP6, COX3, ND3, ND4L, ND4, ND5, and Cytb) were encoded on the H-strand, while ND6 genes encoded on the L-strand. All the protein-coding genes use the initiation codon ATG except COI starts with GTG, which is identical to other vertebrate mtDNA (Gotoh et al. 2009; Thacker and Roje 2009). Most of them have TAA or TAG as the stop codon, whereas four protein-coding genes (COII, ND3, ND4, Cytb) ended with a single T. 12SrRNA (955 bp) was located between tRNA^Phe^ and tRNA^Val^ genes and 16SrRNA (1689 bp) was located between tRNA^Val^ and tRNA^Leu^ genes. The control region, located between tRNA^pro^ and tRNA^Phe^, is 955 bp in length. The origin of light strand replication was located between tRNA^Asn^ and tRNA^Cys^ with 47 bp in length, which is similar to that in other teleostean mitogenomes (Gotoh et al. 2009).

The phylogenetic tree based on the neighbour-joining method was constructed to provide a relationship within Perciformes. The result of the present study supports *A. macropterus* has the closest relationship with *Cheilodipterus quinquelineatus* and *Apogon semilineatus*, highly supported by a bootstrap probability of 99% (Figure 1), which would contribute to the understanding of the phylogeny of Apogonidae.

**Figure 1.**
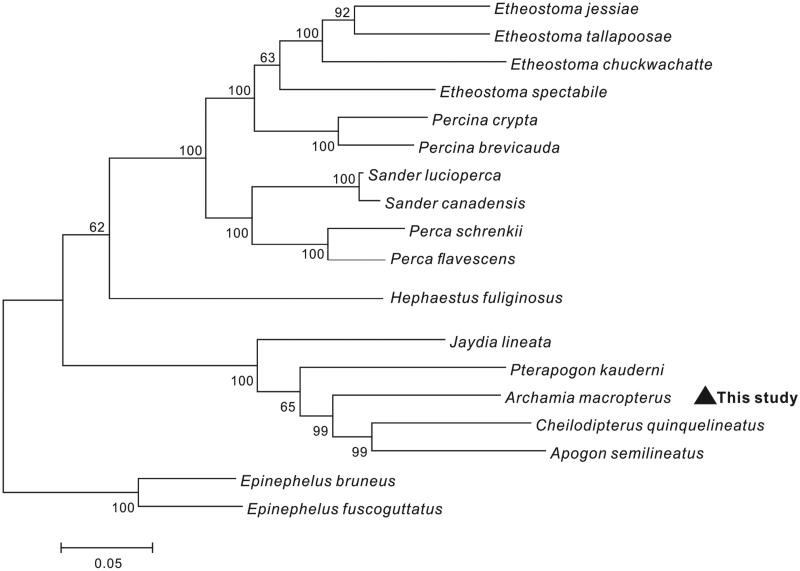
Neighbour-joining tree constructed based on the complete mitogenome of 18 Apogonidae species. The bootstrap values are based on 1000 resamplings. The number at each node is the bootstrap probability. GenBank accession numbers: *Etheostoma jessiae*: NC035944.1, *Etheostoma tallapoosae*: NC035867.1, *Etheostoma chuckwachatte*: NC035943.1, *Etheostoma spectabile*: NC042203.1, *Percina crypta*: NC035945.1, *Percina brevicauda*: NC044075.1, *Sander lucioperca*: NC026533.1, *Sander canadensis*: NC021444.1, *Perca schrenkii*: NC027745.1, *Perca flavescens*: JX629447.1, *Hephaestus fuliginosus*: MH606192.1, *Jaydia lineata*: NC041647.1, *Pterapogon kauderni*: MH049004.1, *Archamia macropterus*: MN066612, *Cheilodipterus quinquelineatus*: NC040863.1, *Apogon semilineatus*: AP005996.1, *Epinephelus bruneus*: NC013820.1 and *Epinephelus fuscoguttatus*: NC020046.1.
